# Supplementary material for: IgG Glycosylation Profiling of Peripheral Artery Diseases with Lectin Microarray
Source: J Clin Med. 2022 Sep 27;11(19):5727. doi: 10.3390/jcm11195727 (PMC9572750; doi:10.3390/jcm11195727)
Supplement: Supplementary file 1 [file jcm-11-05727-s001.zip › revised-Supplementary figure legends.pdf]

Figure S1. Heatmap of association between all lectin microarray results and clinical or laboratory results for LEPAD patients

Rows: samples; columns: lectins and clinical indicators. Preferred binding sugars for lectins was listed with legend. Color key indicates standardized fluorescent intensity for lectins: blue: lowest; red: highest. The heatmap was generated using R software (Version 4.0.2, https://cran.r-project.org/bin/windows/base/old/4.0.2/).

Figure S2. Heatmap of association between all lectin microarray results and clinical or laboratory results for CAS patients

Rows: samples; columns: lectins and clinical indicators. Preferred binding sugars for lectins was listed with legend. Color key indicates standardized fluorescent intensity for lectins: blue: lowest; red: highest. The heatmap was generated using R software (Version 4.0.2, https://cran.r-project.org/bin/windows/base/old/4.0.2/).

Figure S3. Heatmap of significant correlation between selective lectins and laboratory results for LEPAD patients.

R-squared of Pearson correlation is listed and (r^2<0.25) were in blank color.

Figure S4. Heatmap of significant correlation between selective lectins and laboratory results for CAS patients.

R-squared of Pearson correlation is listed and (r^2<0.2) were in blank color.
