# Supplementary material for: IgG Glycosylation Profiling of Peripheral Artery Diseases with Lectin Microarray
Source: J Clin Med. 2022 Sep 27;11(19):5727. doi: 10.3390/jcm11195727 (PMC9572750; doi:10.3390/jcm11195727)

Summary for LEAD lectin blot

Group/subgroup information	lectin	Preferred Sugar	Number of samples
Dyslipidemia vs. normal	Black bean crude	GalNAc	12 in each group
Fontaine severe vs. not severe	Jacalin, AIA	Galβ3GalNAc	9 in each group
Fontaine severe vs. not severe	MNA-M	Mannose	9 in each group
Diabetes vs. normal	PHA-E	Galβ4GlcNAc	9 in each group
Diabetes vs. normal	PHA-L	Galβ4GlcNAc	9 in each group
Hypertension vs. normal	ASA	Mannose	12 in each group
LEAD vs. AAA & CAS	SNA	Sialic acid	7 in each group
LEAD vs. AAA & CAS	ConA	Mannose	8 in each group

Black Bean Crude

blot：

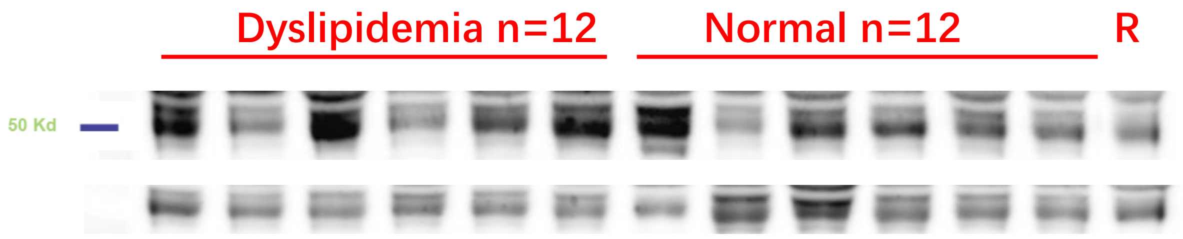

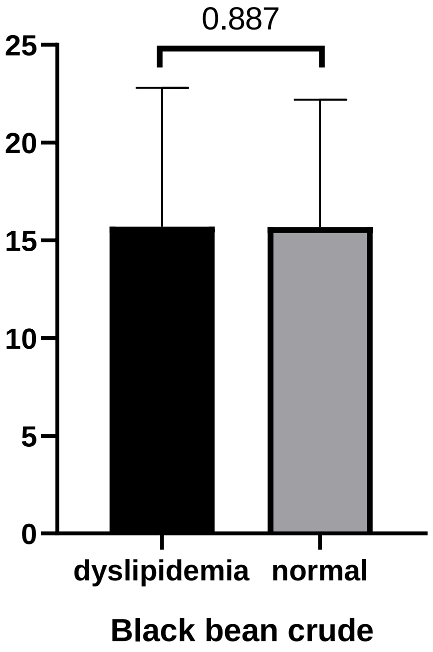

microarray：

group	Lectins	Preferred Sugar	Fc	Mean A	Mean B	P
**dyslipidemia / non**	Black bean crude	GalNAc>lactose >melibiose, galactose, sialic acid	0.570502	7.4723	13.0988	0.038150

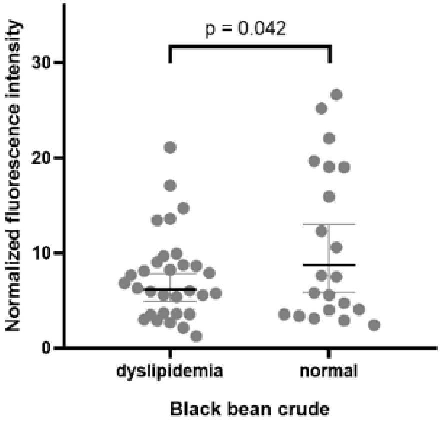

original blot：

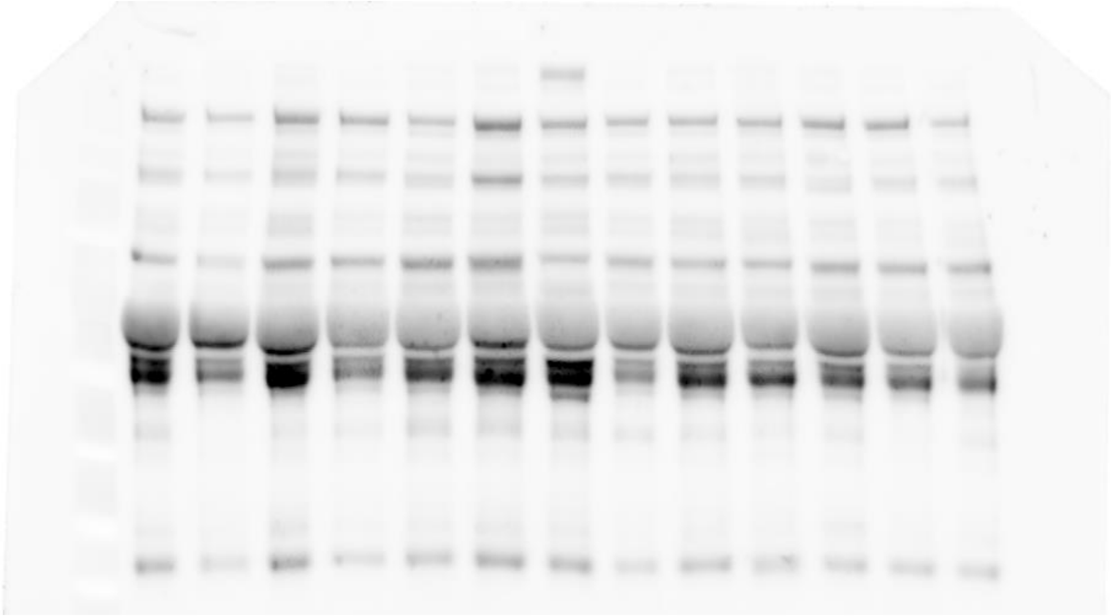

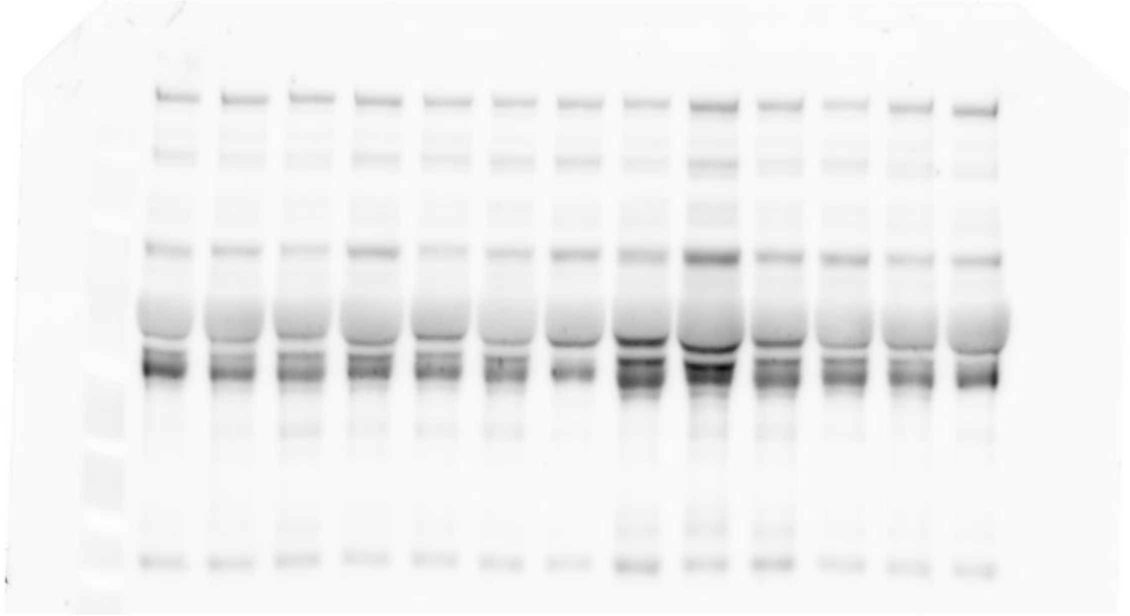

Jacalin-AIA

blot：

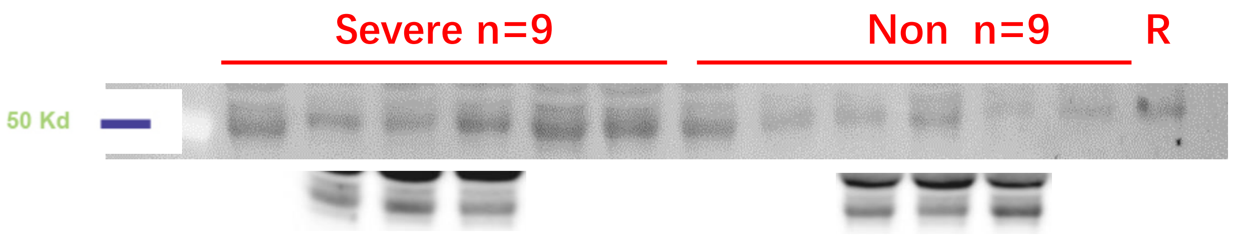

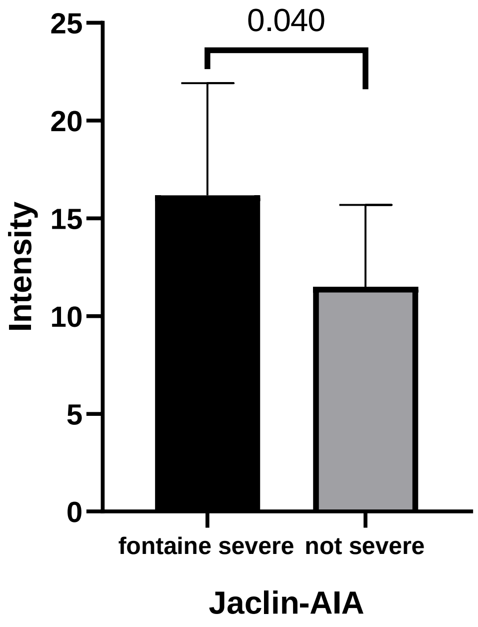

microarray：

group	Lectins	Preferred Sugar	Fc	Mean A	Mean B	P
Severe / non	Jacalin, AIA	Galβ3GalNAc	2.737724	3.274700251	1.19613965	0.015522

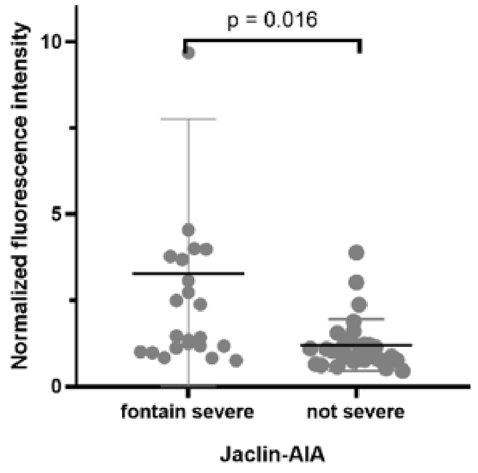

original blot:

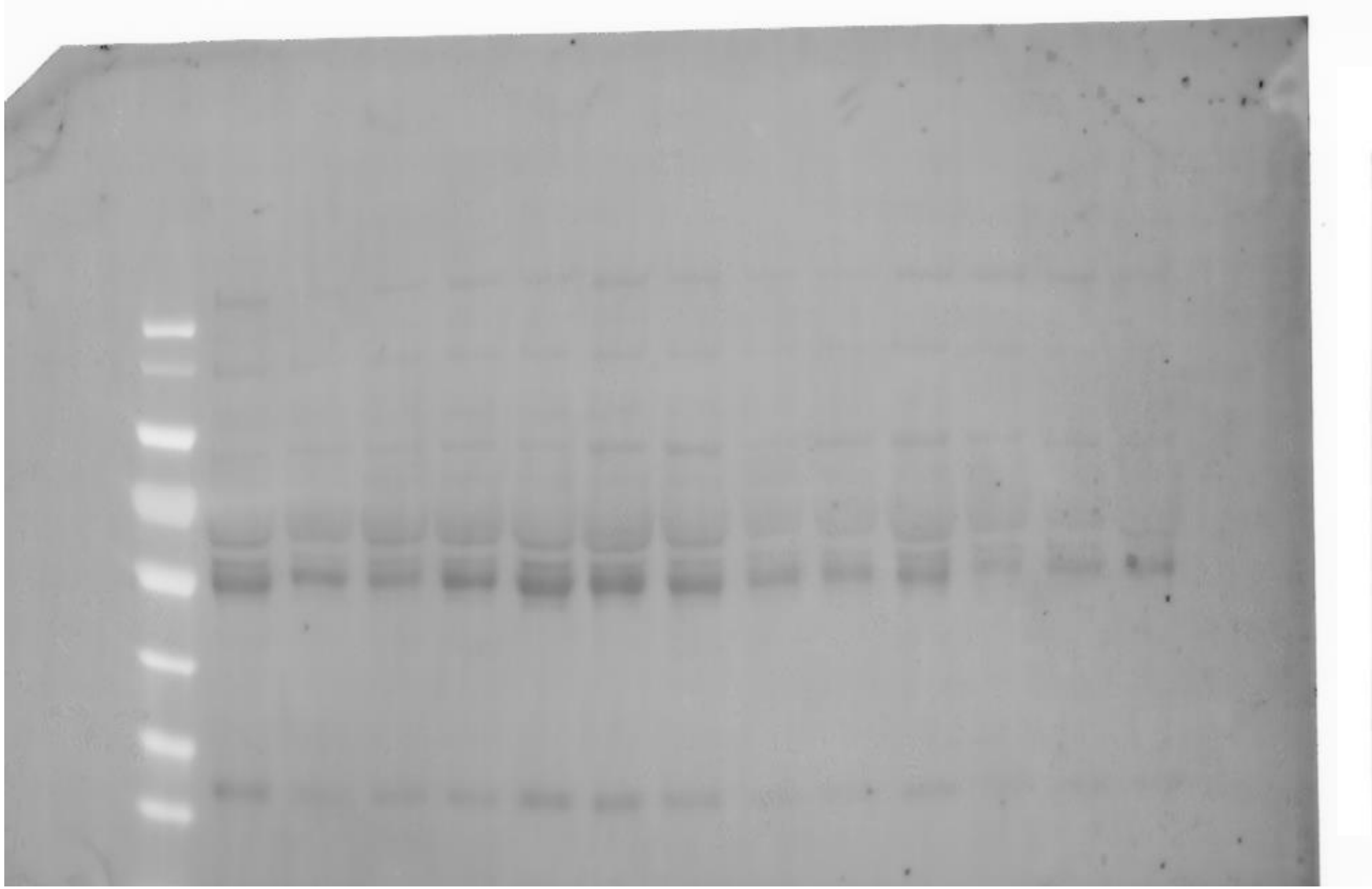

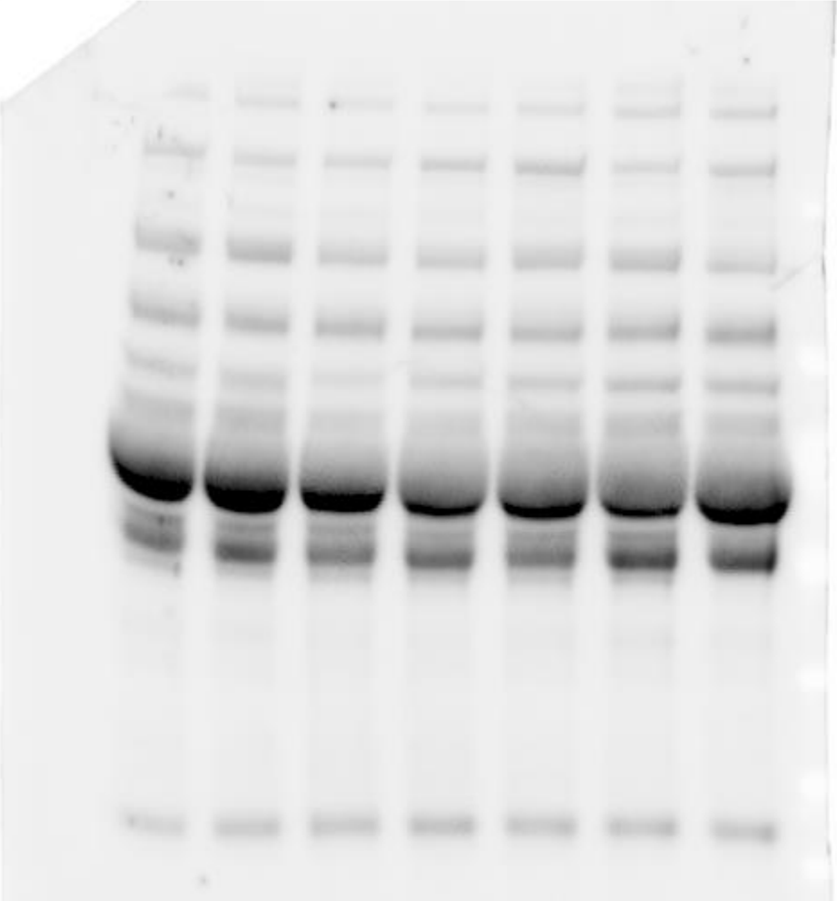

MNA

blot：

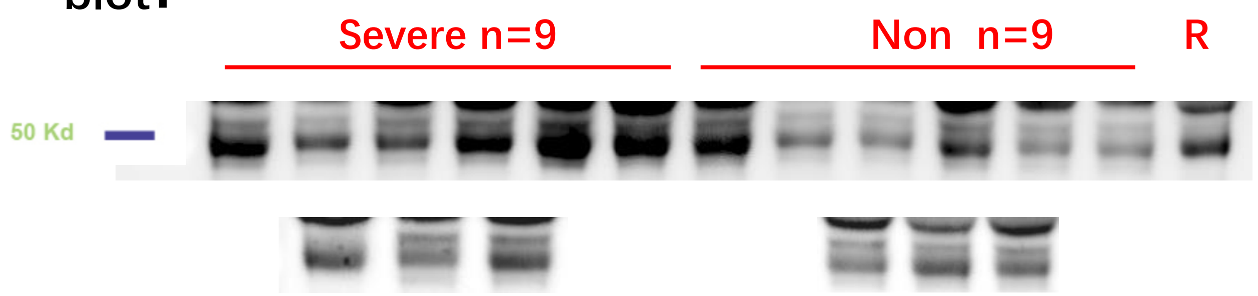

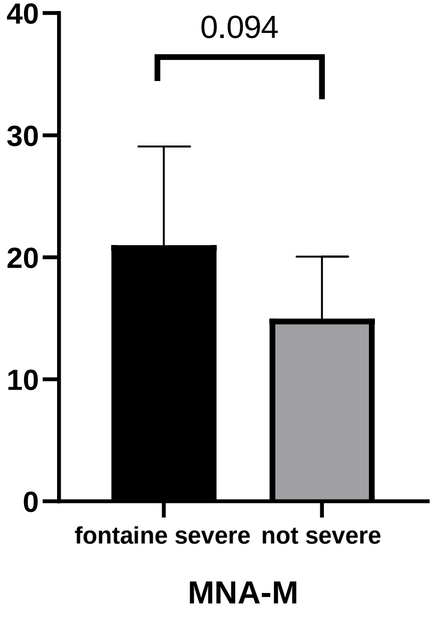

microarray：

group	Lectins	Preferred Sugar	Fc	Mean A	Mean B	P
**Severe / non**	MNA-M	**Man**	1.846778	3.972466494	2.151025016	0.019321

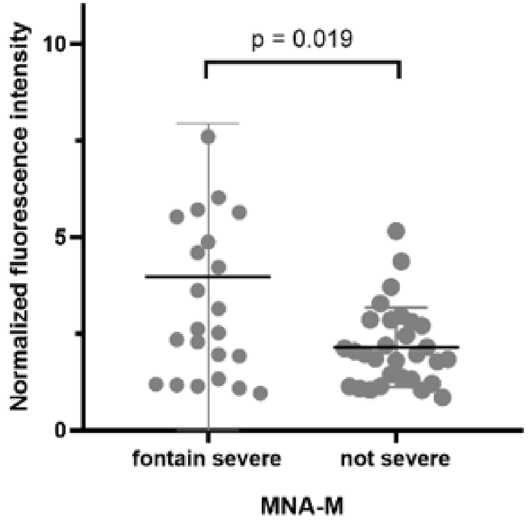

original blot：

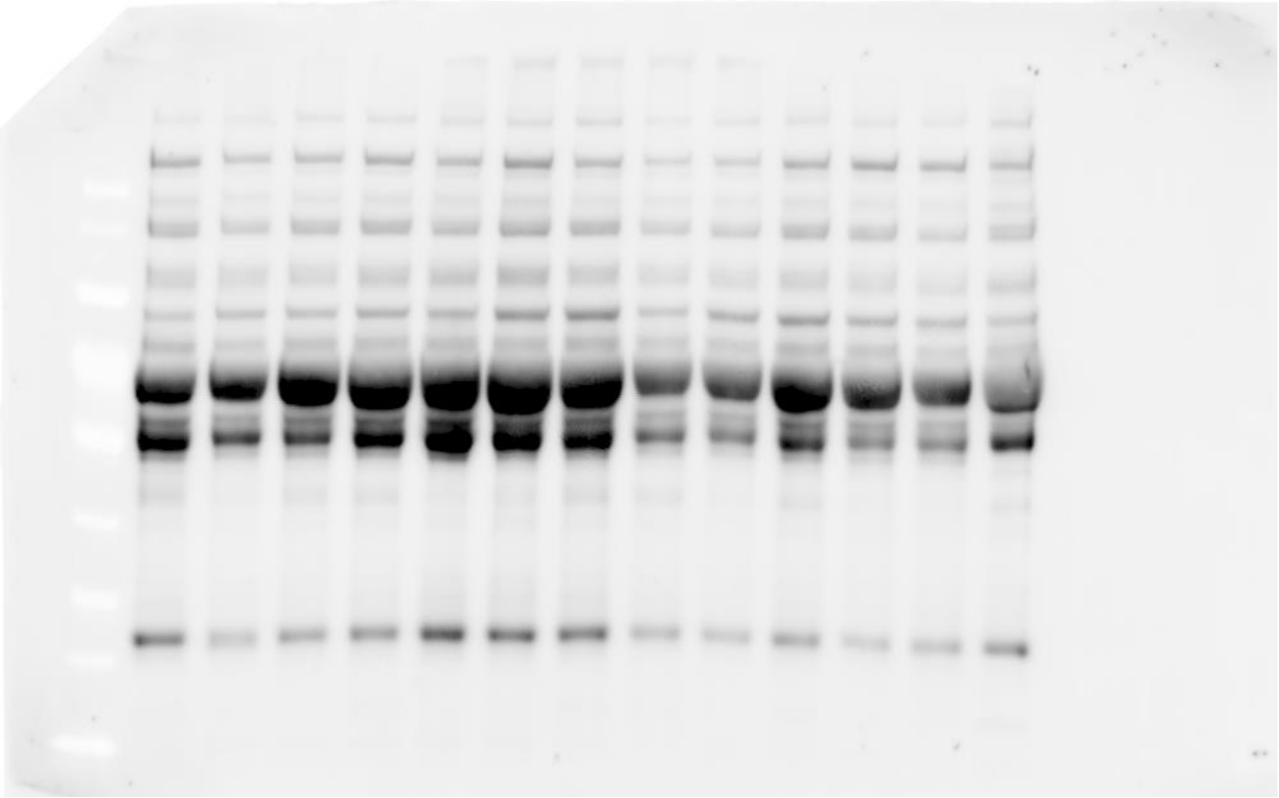

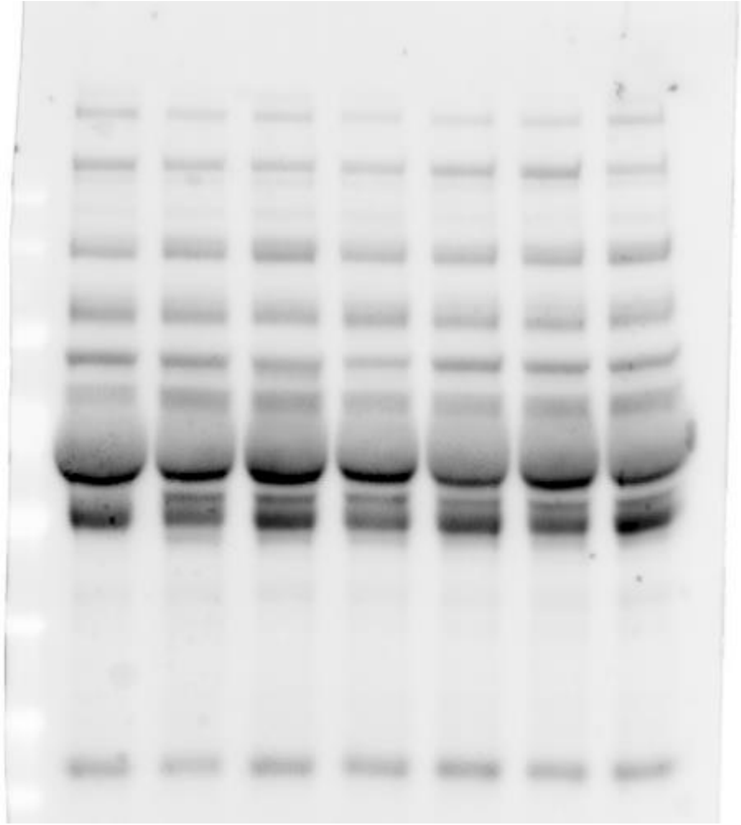

PHA-E

blot:

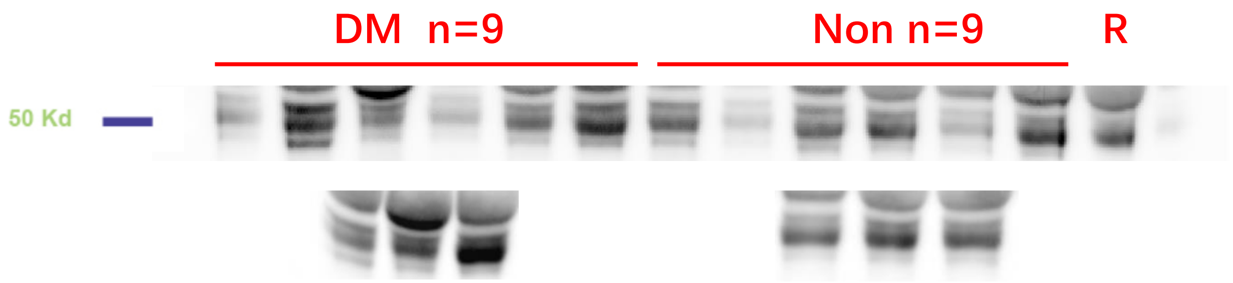

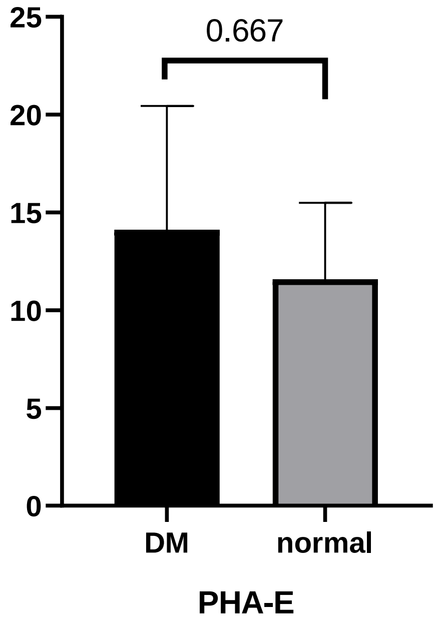

microarray:

group	Lectins	Preferred Sugar	Fc	Mean A	Mean B	P
**DM / non**	PHA-E	Galβ4GlcNAcβ2Manα6(GlcNAcβ4)(GlcNAcβ4Manα3) Manβ4	1.772800	7.596834273	4.285218479	0.012055

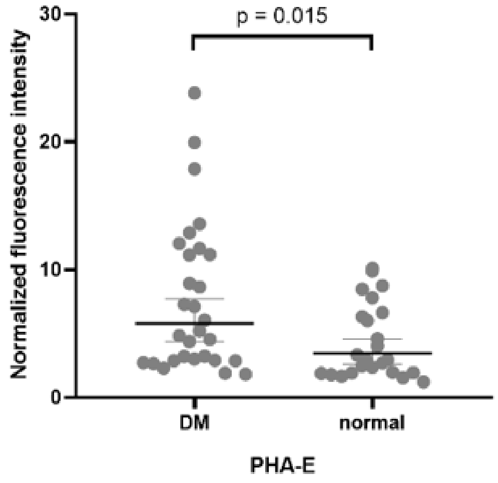

original blot：

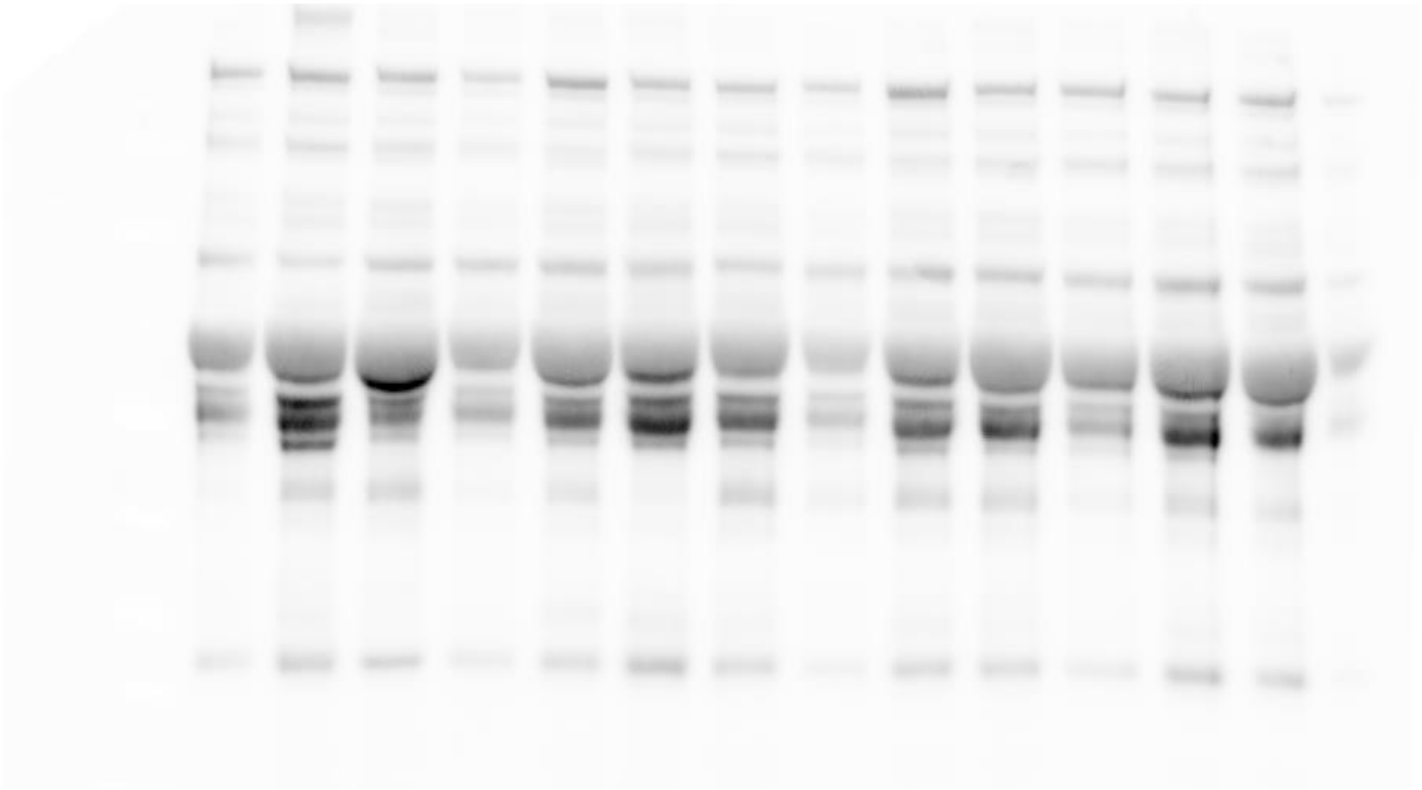

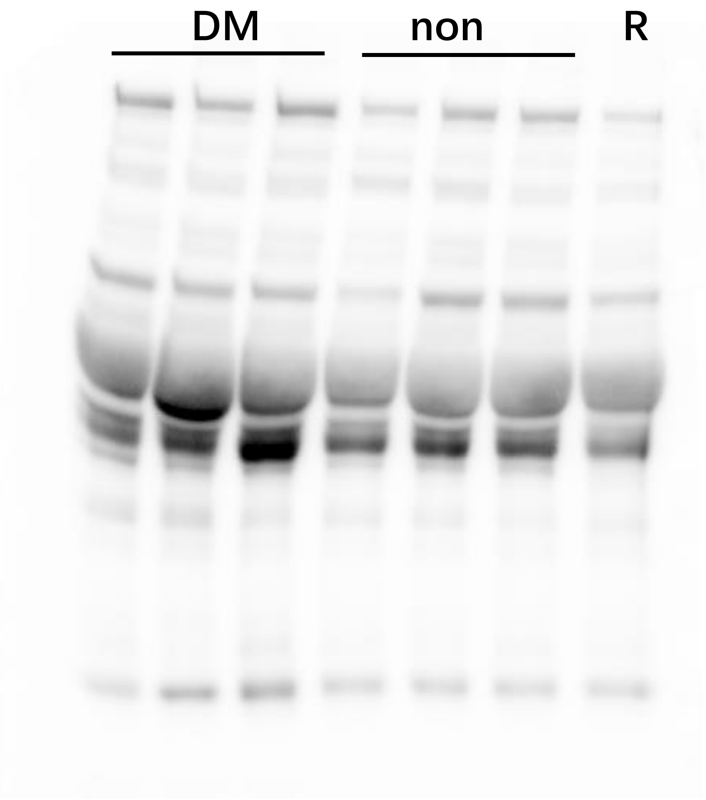

PHA-L

blot：

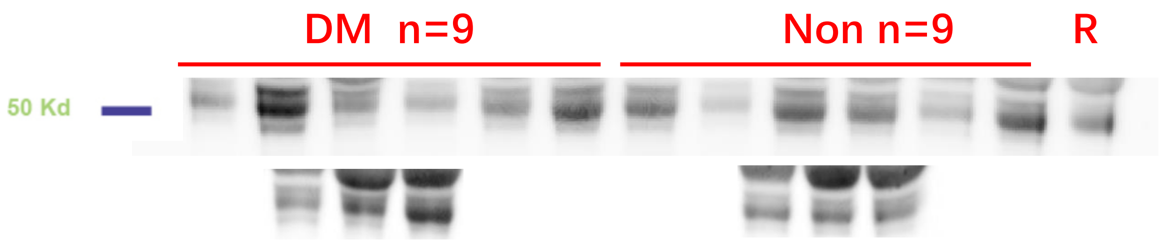

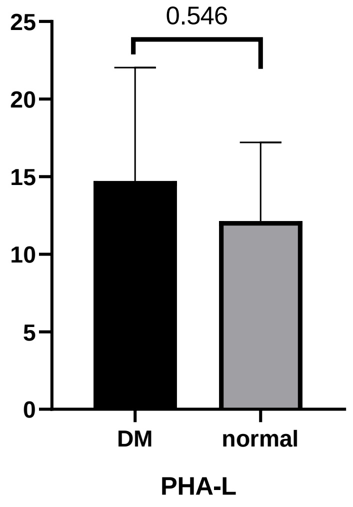

microarray：

group	Lectins	Preferred Sugar	Fc	Mean A	Mean B	P
**DM / non**	PHA-L	Galβ4GlcNAcβ6(GlcNAcβ2Manα3)Manα3	1.772800	9.591664375	4.805694728	0.013331

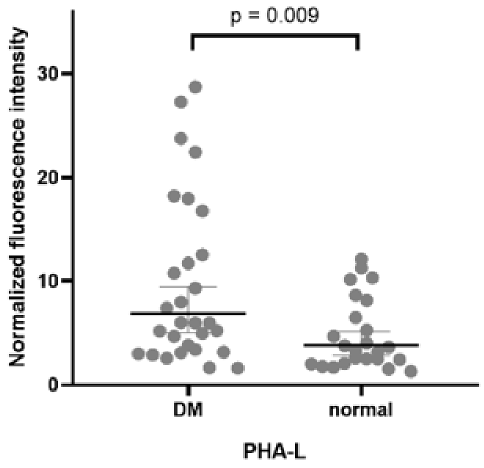

original blot：

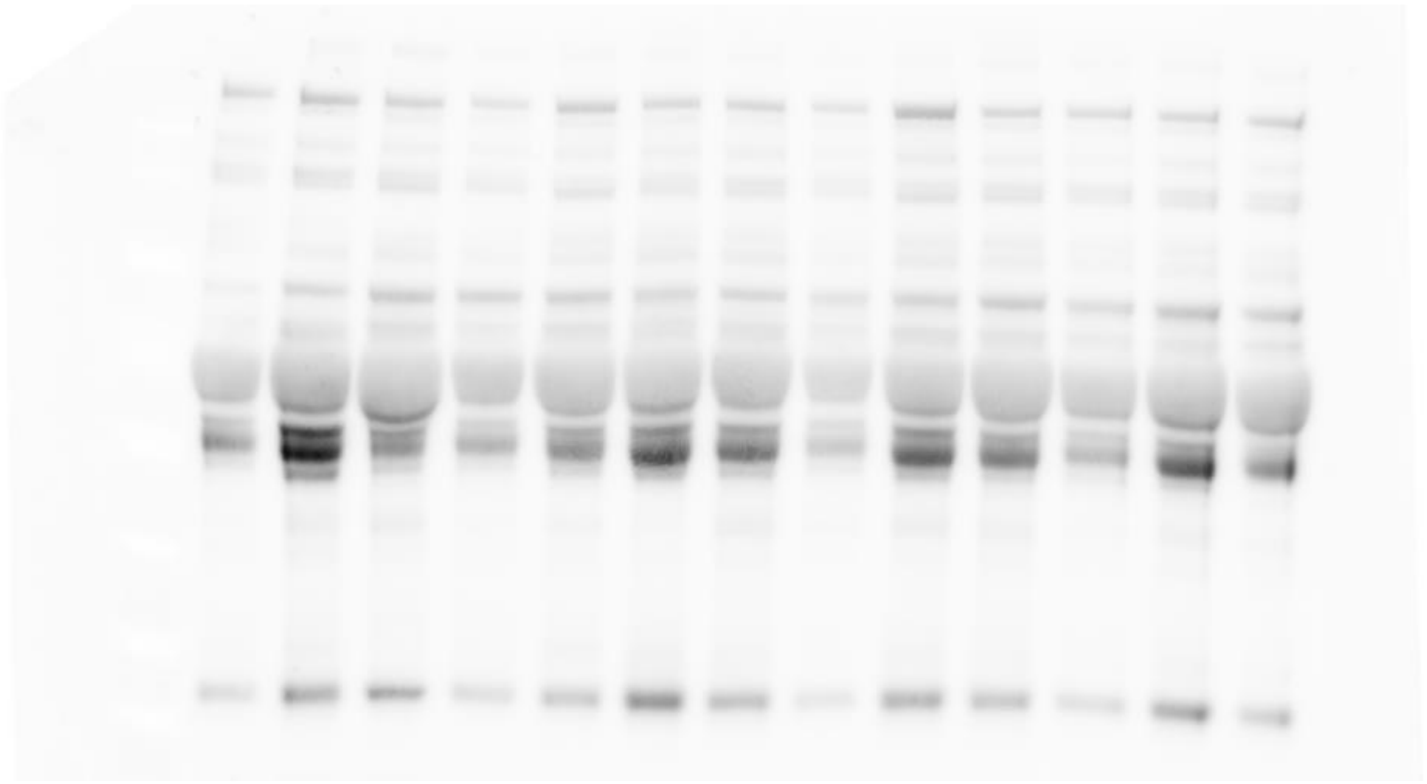

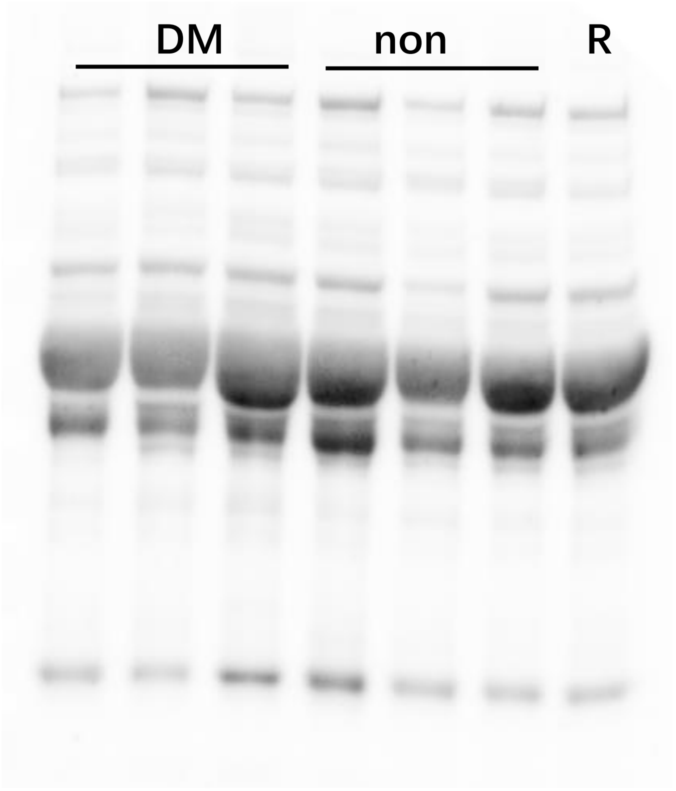

ASA

blot：

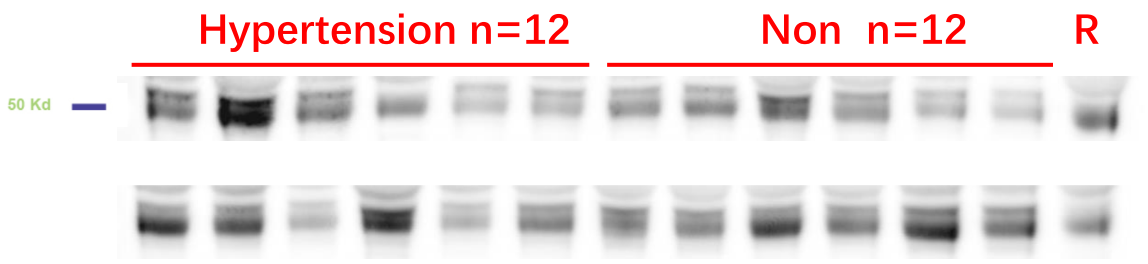

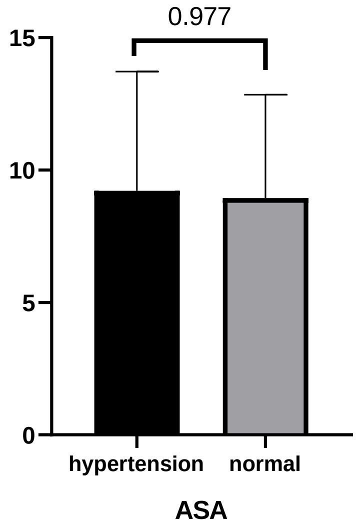

microarray：

group	Lectins	Preferred Sugar	Fc	Mean A	Mean B	P
Hypertension / non	ASA	Man	2.240537	2.049766625	0.914855247	0.010678

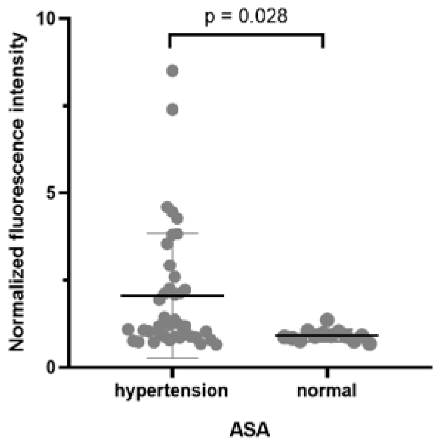

original blot：

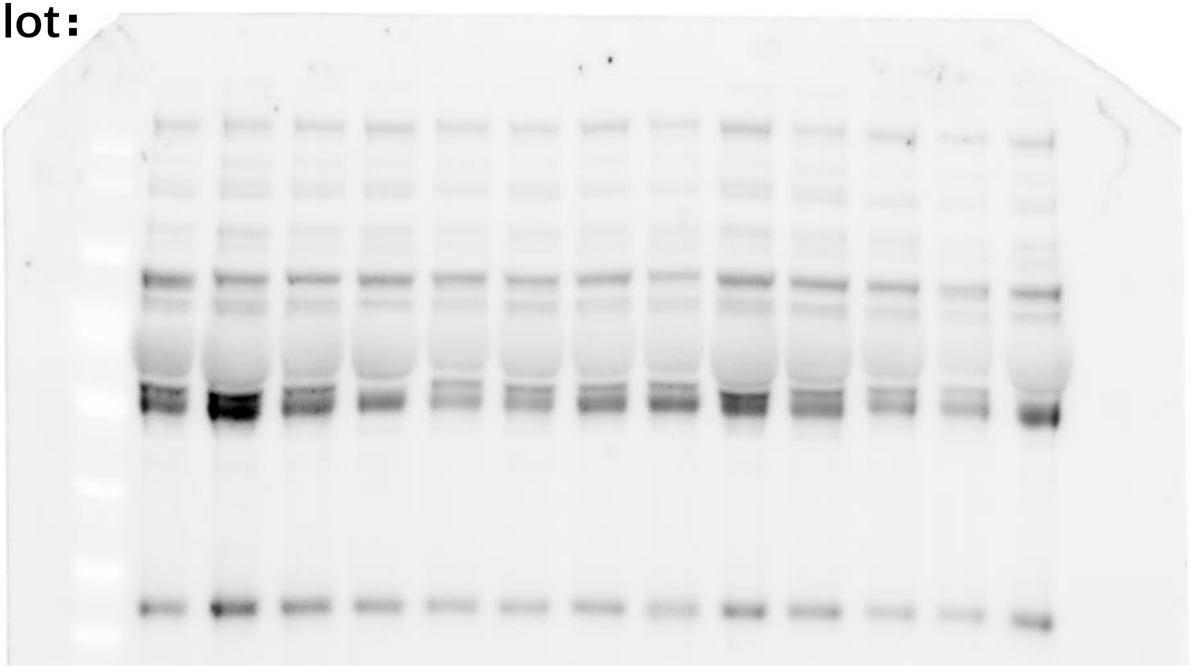

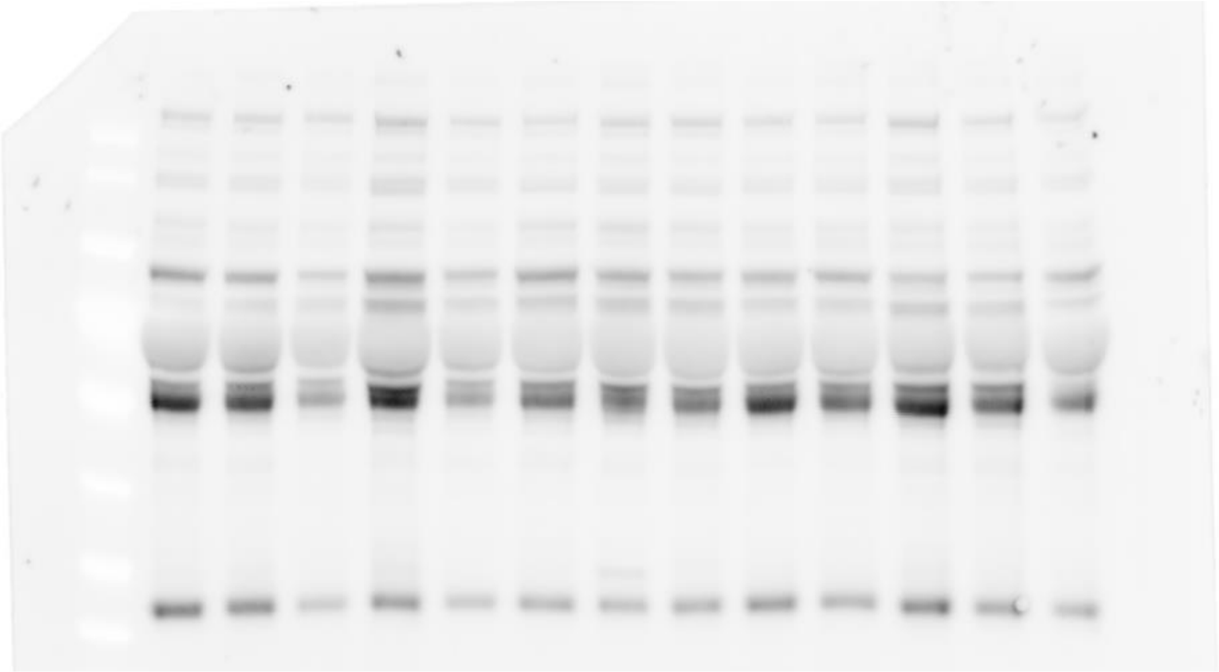

SNA

blot：

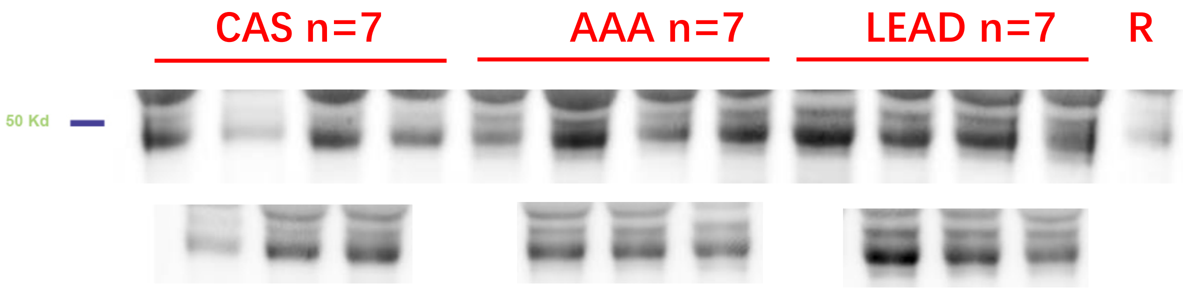

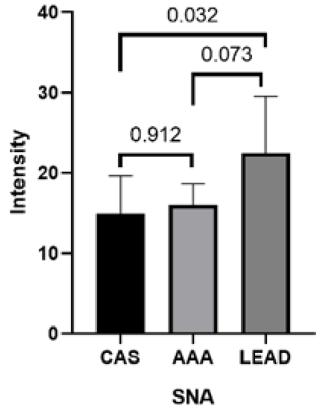

microarray：

group	Lectins	Preferred Sugar	Fc	Mean A	Mean B	P
**AAA / CAS**	SNA	Neu5Acα6Gal/ GalNAc	1.351201	3.33848535 9	2.47075431 2	0.0085 271
**CLI / CAS**	ConA<br>SNA	αMan,αGlc<br>Neu5Acα6Gal/ GalNAc	1.496571172<br>1.301025978	6.94881244 1<br>3.21451554 6	4.64315534 8<br>2.47075431 2	0.0024 862<br>0.0181 746

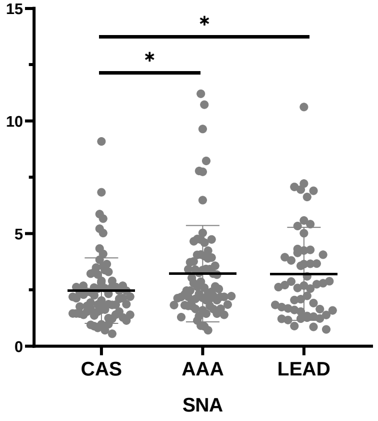

original blot：

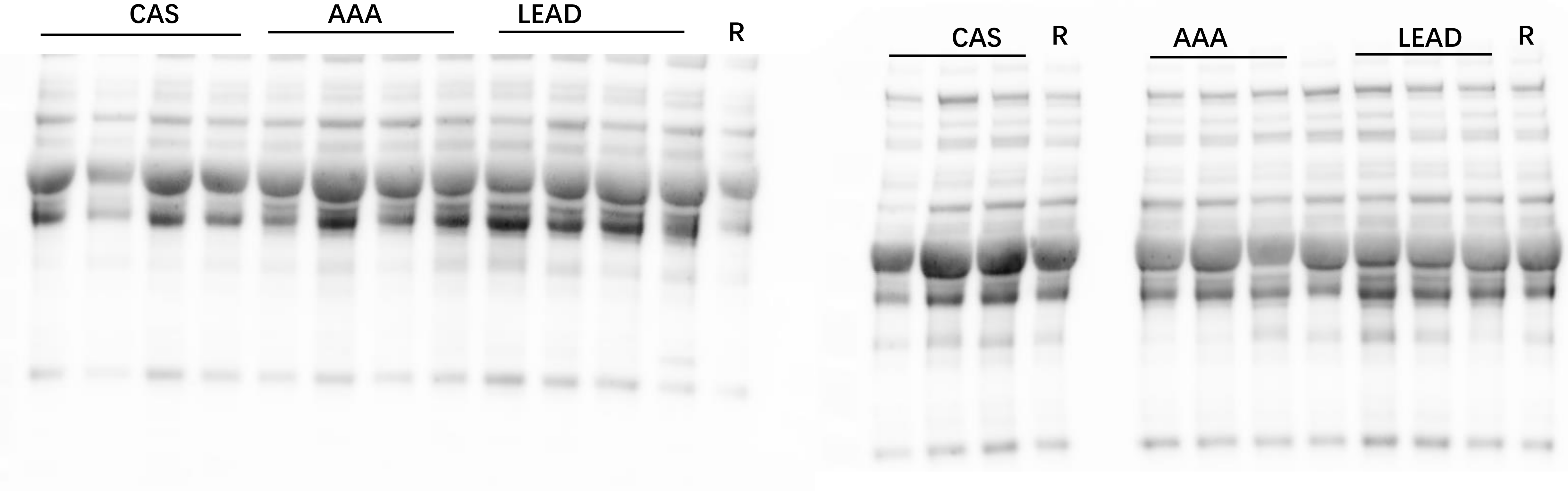

Con A

blot：

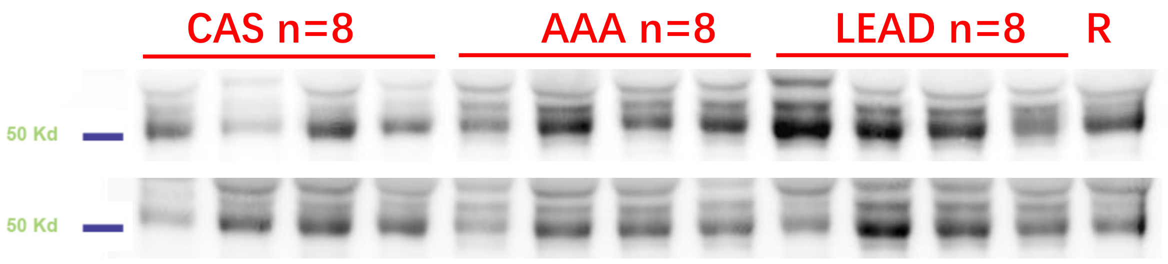

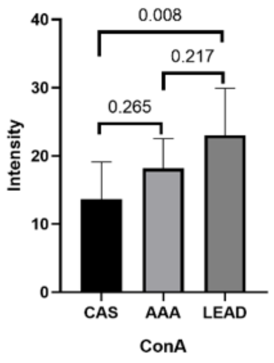

microarray：

group	Lectins	Preferred Sugar	Fc	Mean A	Mean B	P
**CLI / CAS**	ConA<br>SNA	αMan,αGlc<br>Neu5Acα6Gal/<br>GalNAc	1.496571172<br>1.301025978	6.94881244<br>1<br>3.21451554<br>6	4.64315534<br>8<br>2.47075431<br>2	0.0024<br>862<br>0.0181<br>746

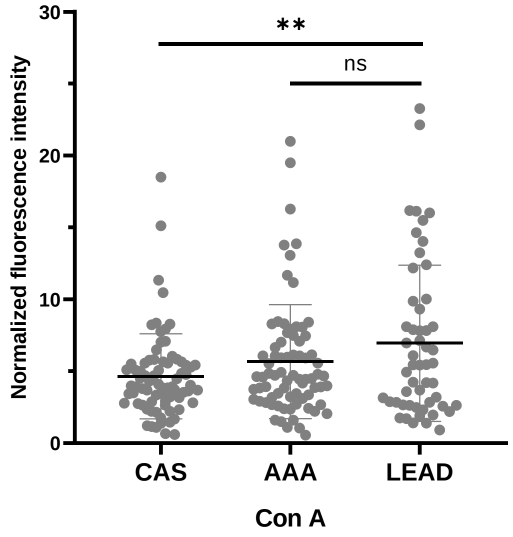

original blot：

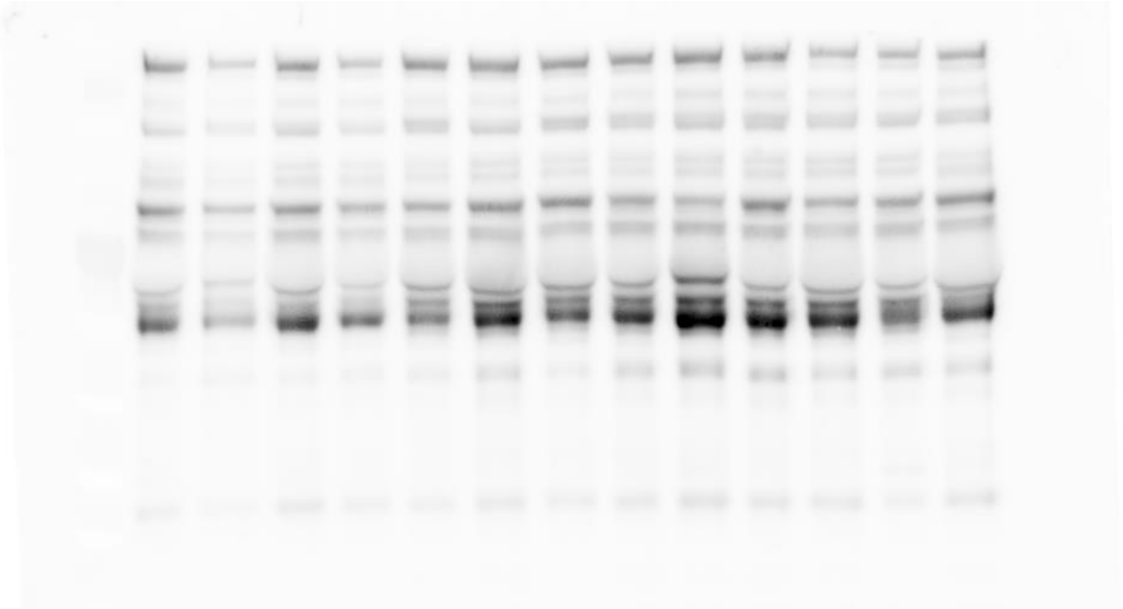

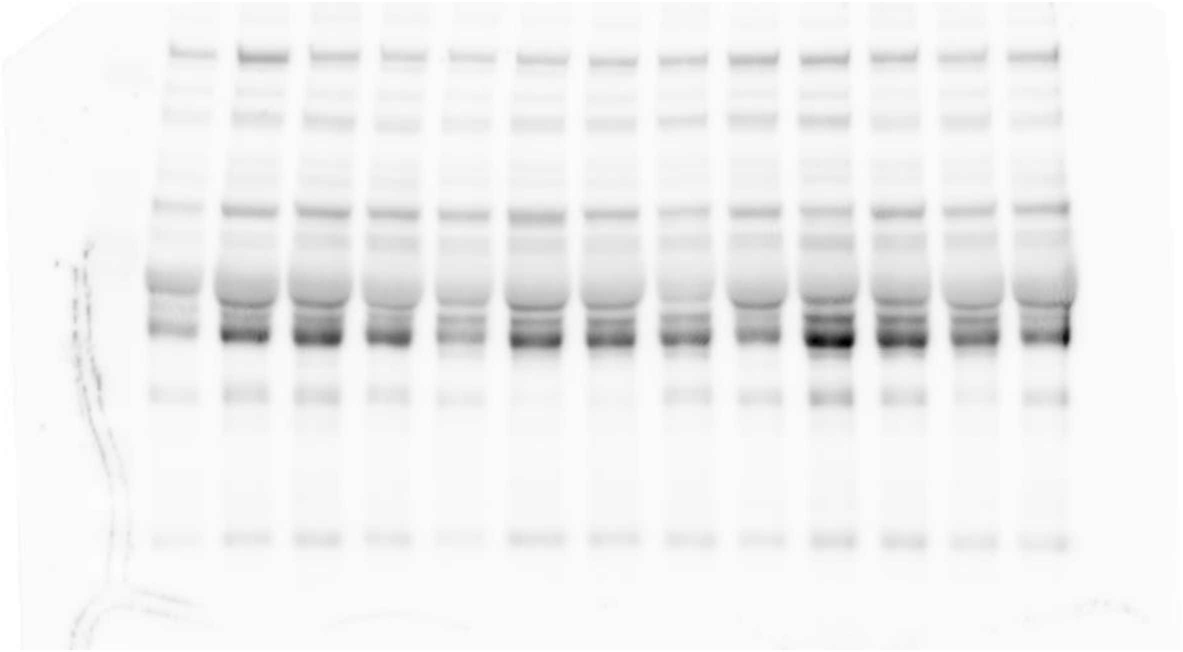

Supplement: Supplementary file 1 [file jcm-11-05727-s001.zip › Supplemental-lectin blot_original blot.pdf]
