# Supplementary figures and images for: IgG Glycosylation Profiling of Peripheral Artery Diseases with Lectin Microarray
Source: J Clin Med. 2022 Sep 27;11(19):5727. doi: 10.3390/jcm11195727 (PMC9572750; doi:10.3390/jcm11195727)

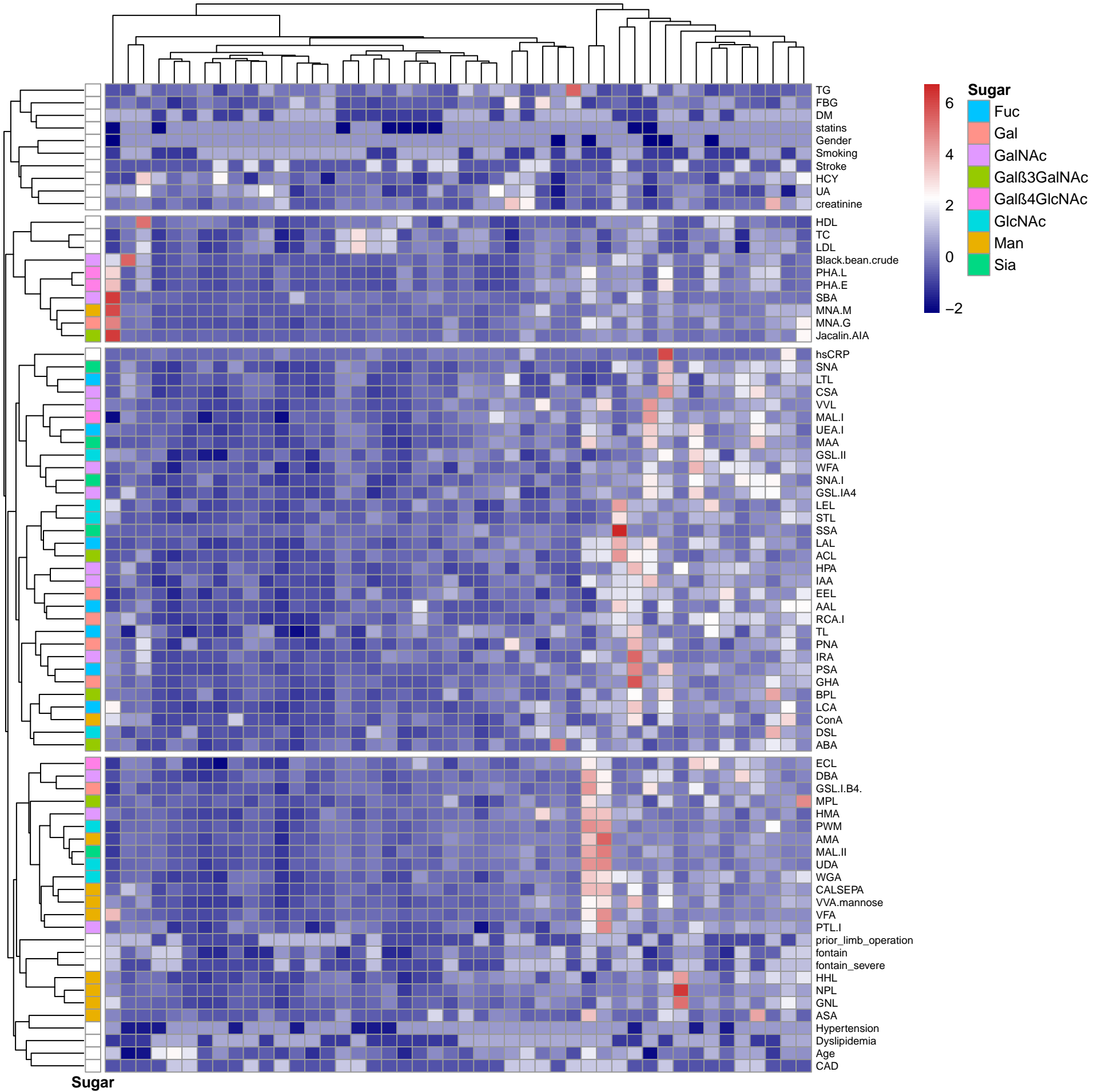

Supplement: Supplementary file 1 [file jcm-11-05727-s001.zip › Figure S1-LEPAD-heatmap-all.pdf]

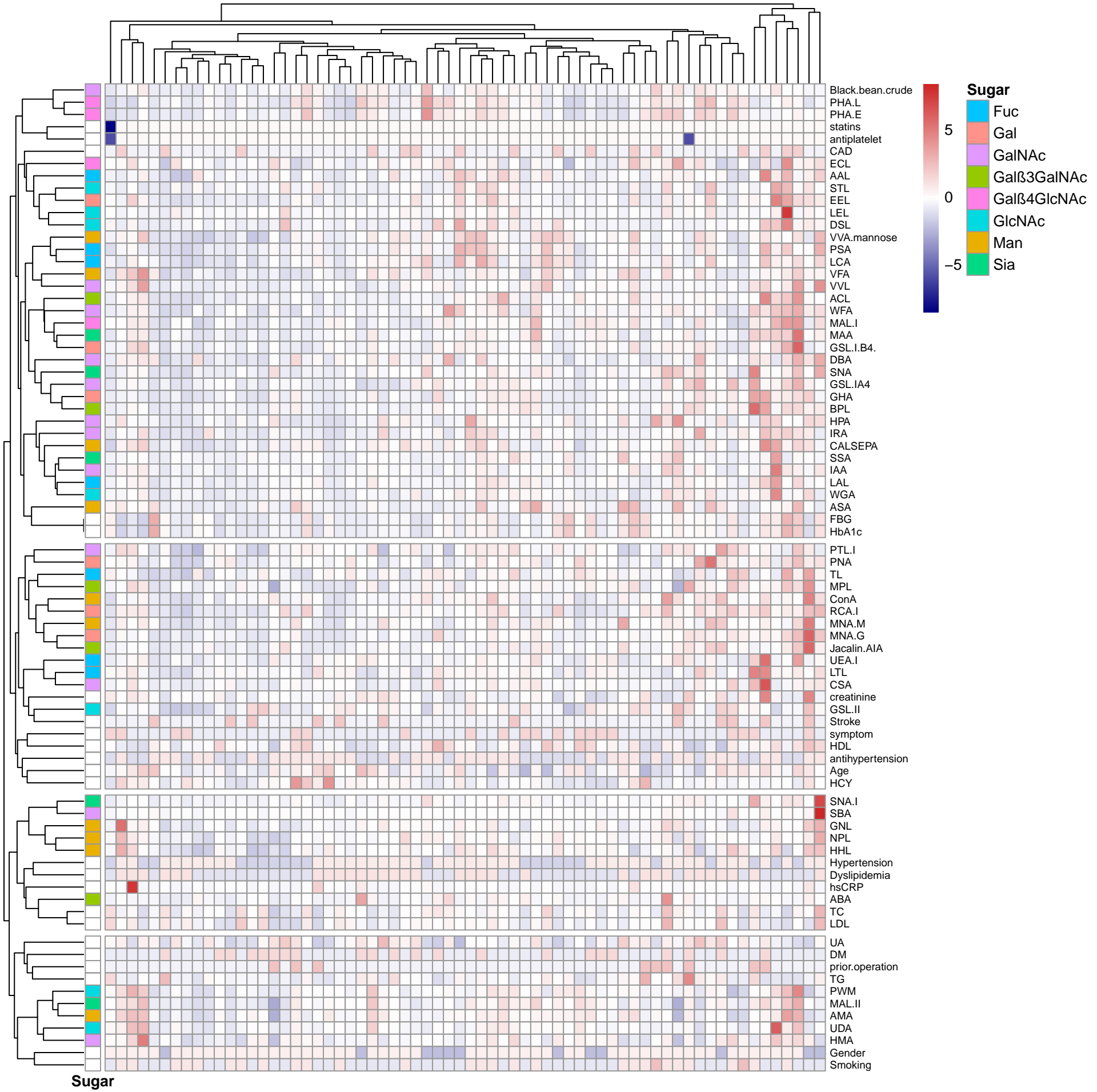

Supplement: Supplementary file 1 [file jcm-11-05727-s001.zip › Figure S2-CAS-heatmap-all.pdf]

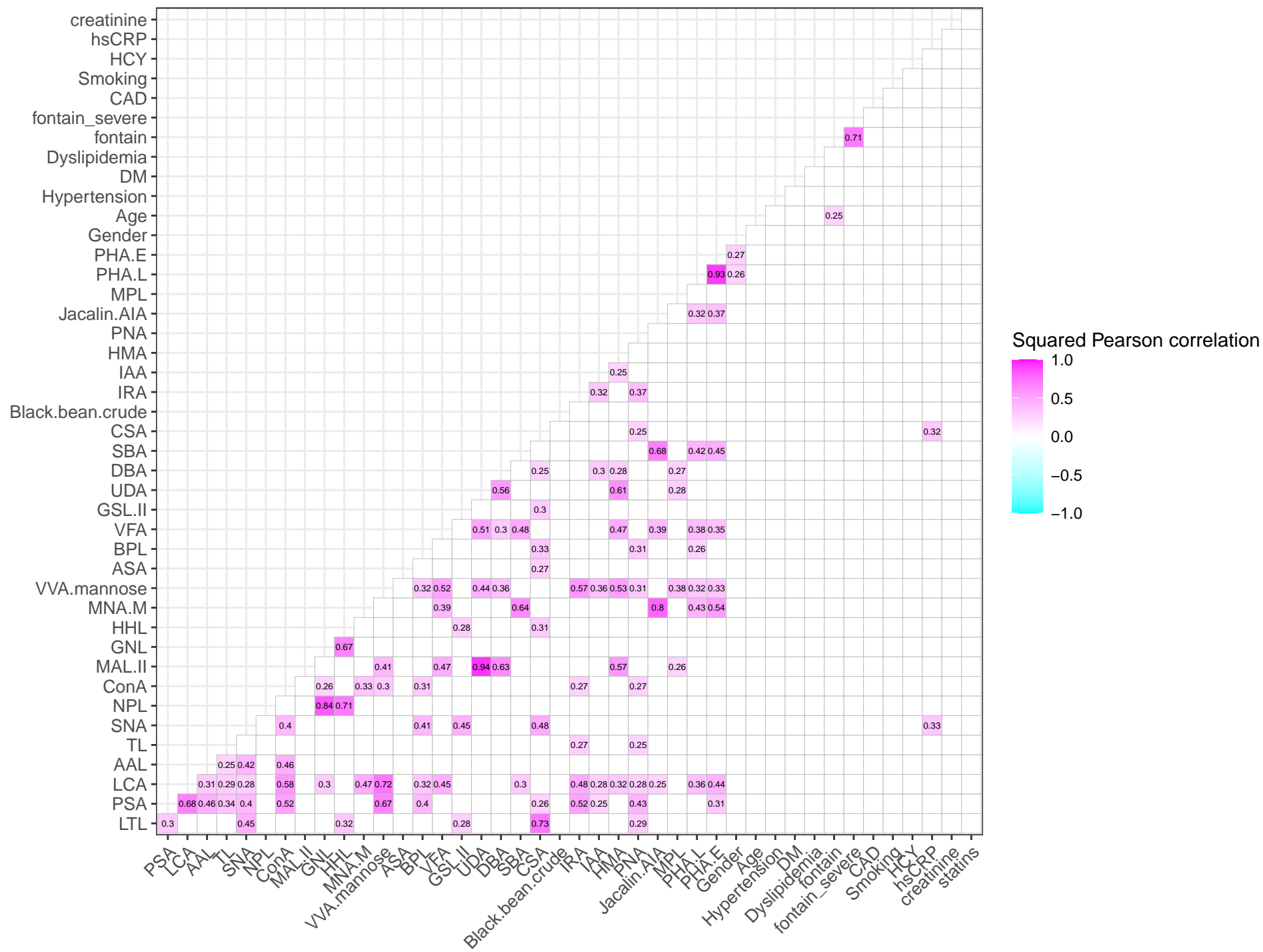

Supplement: Supplementary file 1 [file jcm-11-05727-s001.zip › Figure S3 LEPAD.pdf]

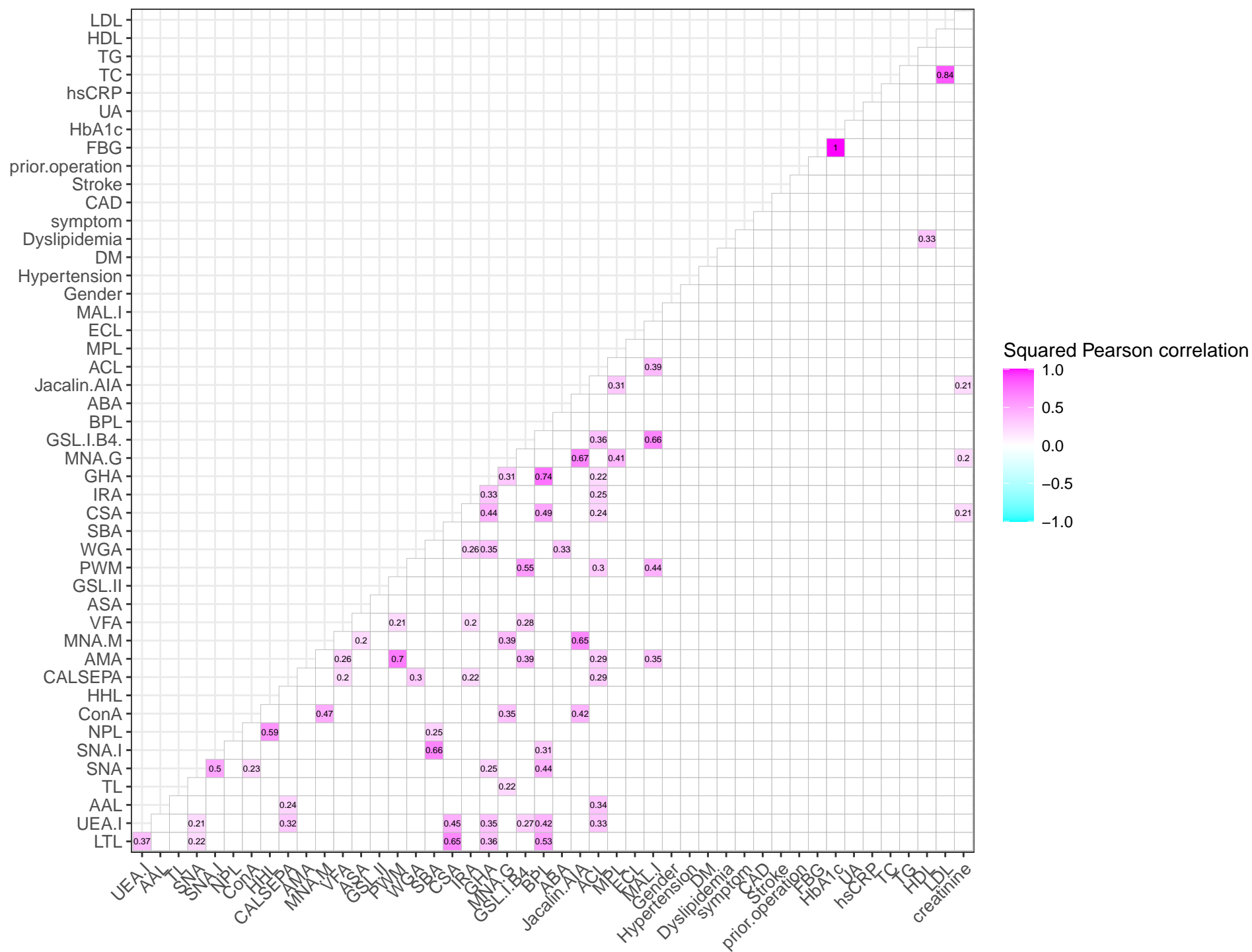

Supplement: Supplementary file 1 [file jcm-11-05727-s001.zip › Figure S4-squared CAS.pdf]
